# Comparison of tumour and serum specific microRNA changes dissecting their role in pancreatic ductal adenocarcinoma: a meta-analysis

**DOI:** 10.1186/s12885-019-6380-z

**Published:** 2019-12-03

**Authors:** Bishnupriya Chhatriya, Moumita Mukherjee, Sukanta Ray, Piyali Sarkar, Shatakshee Chatterjee, Debashis Nath, Kshaunish Das, Srikanta Goswami

**Affiliations:** 1 National Institute of Biomedical Genomics, Kalyani, West Bengal India; 2 School of Digestive and Liver Diseases, Institute of Post Graduate Medical Education and Research, Kolkata, West Bengal India; 3 Present Address: Tata Medical Centre, Kolkata, West Bengal India; 4 Indira Gandhi Memorial Hospital, Agartala, Tripura India

**Keywords:** Pancreatic ductal adenocarcinoma, Serum, Tumour, microRNA, Meta-analysis

## Abstract

**Background:**

Pancreatic ductal adenocarcinoma (PDAC) is considered as one of the most aggressive cancers lacking efficient early detection biomarkers. Circulating miRNAs are now being considered to have potency to be used as diagnostic and prognostic biomarkers in different diseases as well as cancers. In case of cancer, a fraction of the circulating miRNAs is actually derived from the tumour tissue. This fraction would function as stable biomarker for the disease and also would contribute to the understanding of the disease development. There are not many studies exploring this aspect in pancreatic cancer and even there is not much overlap of results between existing studies.

**Methods:**

In order to address that gap, we performed a miRNA microarray analysis to identify differentially expressed circulating miRNAs between PDAC patients and normal healthy individuals and also found two more similar datasets to perform a meta-analysis using a total of 182 PDAC patients and 170 normal, identifying a set of miRNAs significantly altered in patient serum. Next, we found five datasets studying miRNA expression profile in tumour tissues of PDAC patients as compared to normal pancreas and performed a second meta-analysis using data from a total of 183 pancreatic tumour and 47 normal pancreas to detect significantly deregulated miRNAs in pancreatic carcinoma. Comparison of these two lists and subsequent search for their target genes which were also deregulated in PDAC in inverse direction to miRNAs was done followed by investigation of their role in disease development.

**Results:**

We identified 21 miRNAs altered in both pancreatic tumour tissue and serum. While deciphering the functions of their target genes, we characterized key miR-Gene interactions perturbing the biological pathways. We identified important cancer related pathways, pancreas specific pathways, AGE-RAGE signaling, prolactin signaling and insulin resistance signaling pathways among the most affected ones. We also reported the possible involvement of crucial transcription factors in the process.

**Conclusions:**

Our study identified a unique meta-signature of 21 miRNAs capable of explaining pancreatic carcinogenesis and possibly holding the potential to act as biomarker for the disease detection which could be explored further.

## Background

Pancreatic cancer is one of the most aggressive cancers with very low 5 year survival rate (6%) [1]. One of the major reasons for the dismal outcome is the fact that in most of the cases the disease is detected at an advanced stage. The situation is complicated further due to the lack of efficient early detection methods. Circulating biomarkers are generally preferred as screening tools along with various imaging methods and CA 19–9 is the only circulating biomarker being used for pancreatic ductal adenocarcinoma (PDAC). However, its sensitivity and specificity is not good enough for its application as early detection biomarker of PDAC [2]. Recently, circulating nucleic acids like tumor DNA, mRNA and non-coding RNAs (ncRNAs) are being explored for their biomarker potential in different diseases as well as in cancer [3]. microRNAs (miRNAs) are most well studied among all the ncRNAs. They are ~ 22 nt long single stranded ncRNAs, capable of post-transcriptional regulation of gene expression. Altered expression of miRNAs in tissues followed by subsequent deregulation of their target genes has been implicated to development and progression of various cancers. Further exploration have established that miRNAs are secreted into body fluid, primarily, being packaged into exosomes [4]. Therefore, circulating or exosome bound miRNAs have also been implicated to carry organ or disease specific signatures and many studies have been initiated to evaluate the potential of circulating miRNAs to function as non-invasive biomarker for specific diseases.

Similar efforts have been made in pancreatic cancer too. Independent studies have identified miR-21, miR-210, miR-155, miR-200a/b, miR-196a/b etc. in serum or plasma of PDAC patients as well as different miRNAs in tumour tissues [5–7]. However, major drawbacks of all these studies are that there are not much of common miRNAs between them and not all the studies have compared the serum/ plasma expression with the corresponding changes in pancreatic tumour tissue. Furthermore, small sample sizes of individual studies are also a problem. Therefore, we decided to perform meta-analysis where results from multiple individual studies were compared in order to increase the statistical power. We have performed a serum miRNA microarray experiment ourselves to find out relative miRNA expression changes between normal individuals and PDAC patients in our cohort and results of which has also been included in the meta-analysis along with other datasets showing similar results. We have also carried out a second meta-analysis to identify the key miRNA changes present in pancreatic tumor tissue and compared the results of these two meta-analyses to finally derive the PDAC specific meta-signature of miRNAs in serum of these patients. Exosomal contribution to the serum miRNA pool was further assessed by comparing our findings to already available results in databases like Exocarta and miRandola [8, 9]. Finally, considering the importance of these miRNAs being altered in both tumor tissue and in serum, we wanted to investigate the effect or the consequence of these alterations and eventually identified target genes and relevant pathways through which the pathogenesis could be explained.

## Methods

### Patients and blood collection

The pancreatic ductal adenocarcinoma patients were recruited from School of Digestive and Liver Diseases, Institute of Post Graduate Medical Education and Research (IPGME&R) and the age matched normal individuals were healthy volunteers recruited from Indira Gandhi Memorial Hospital. Approval from Institutional Ethics Committee was taken from all the Institutions and written informed consent was obtained from all the recruits prior to the study. From all the recruits 5 ml of peripheral venous blood was collected in vacutainer-serum tubes (BD, USA) and processed within 1 h after blood sampling. After clot has formed, the tubes were centrifuged at 1500×g for 10 min and supernatant was transferred into a new tube and centrifuged again at 2000×g for 10 min. Serum was collected and stored frozen in aliquots at − 80 °C. Only samples without any indication of haemolysis at all stages of serum preparation were used in further study.

### Serum RNA isolation and miRNA microarray

Total serum RNA enriched for small RNAs was isolated using miRNAEasy kit from Qiagen using glycogen method and Affymetrix miRNA 4.0 platform was used for microarray. Probe hybridization was done at 48 °C for 16 h at 60 rpm. Affymetrix 3000 7G scanner was used for data acquisition.

### Selection of datasets

Datasets were searched in GEO and ArrayExpress using the keywords ‘Pancreatic Cancer’, ‘miRNA profiling’ and ‘microarray’. Datasets using ‘serum’ as source were included in “Serum” datasets group and similarly datasets using ‘tissue’ as source were included in “Tissue” datasets group. In “Tissue” datasets group, only those datasets were selected where the tissue samples were fresh frozen by liquid N_2_ or stabilized by lysis buffer. Datasets were excluded if data was obtained from some other source e.g. blood, saliva etc. From the datasets, ‘Pancreatic Cancer’ samples were defined as ‘cases’ and ‘healthy control’ samples were defined as ‘controls’.

### Processing of datasets

Datasets were processed individually and unsupervised analysis was done using R. Dataset processing included normalization by appropriate method, if raw data was used. In case of processed data above mentioned step was skipped. Unsupervised hierarchical clustering and PCA was done to remove the outlier samples.

### Meta-analysis

After the initial processing, “Rankproduct” method was used to do the meta-analysis using R bioconductor package ‘RankProd’ [10–12]. This package has the capacity to combine datasets from different origins (meta-analysis) to increase the power of the identification. The expression data obtained after normalization and removal of outlier for each datasets were merged to form a combined expression data file and the origin and disease status of the samples were specified in another file. Both the files were used as input files using RankProd to obtain DemiRs based on percentage of false prediction (PFP). A cutoff of PFP < 0.05 was used. This method was applied for group of serum and tissue datasets separately to obtain list of differentially regulated miRNAs in serum and tissue respectively.

### DemiR selection

**Fig. 1:**
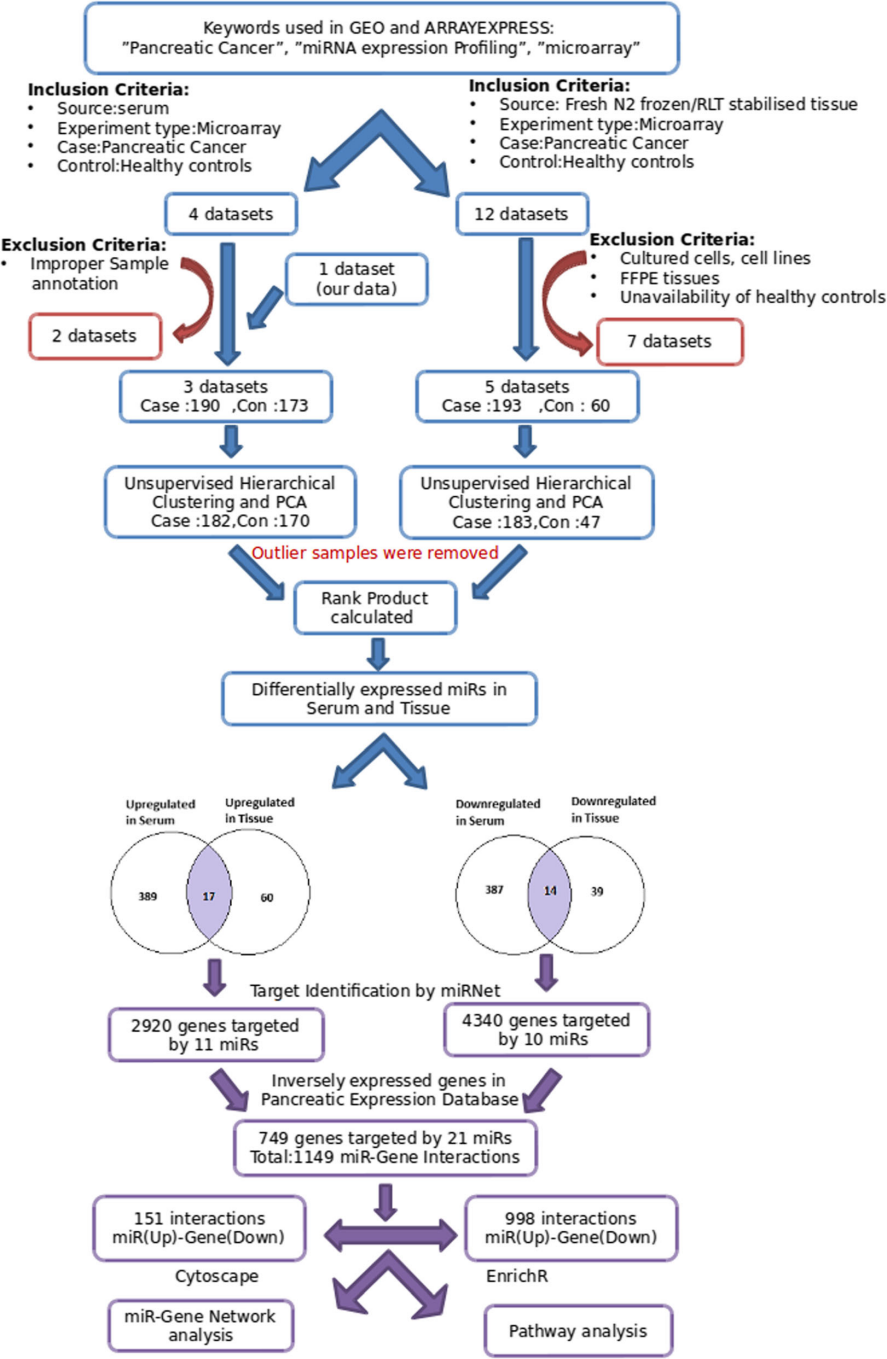
Schematic flowchart representing the study design followed in the study to identify serum specific miRNAs altered in PDAC

The differentially expressed miRNAs obtained in serum and tissue were then compared with each other using Venny v2.1 [13] and the miRNAs which were present in both could be believed to be coming from pancreatic tissue and then secreted in serum and were selected for further studies. Selected miRNAs were further subjected to another criterion of being reported in Pancreatic Expression Database (PED). It is a database which catalogues various biomolecules like RNA, protein associated with pancreatic cancer as reported in published literature [14, 15]. We also checked whether the selected miRNAs were secreted in exosome using databases like Exocarta and miRandola, as described in Fig. 1. Exocarta is an exosome database which enlists the contents of exosomes across different species [8, 16–18]. miRandola is a database containing manually curated information regarding different extracellular circulating non-coding RNA types [9].

### Target identification and selection

Experimentally validated targets for the selected miRNAs were identified using miRNet [19, 20]. It is a web-tool which provides statistical and functional support for miRNA studies. Based on the expression status of target genes, a hypergeometric test was done to identify the miRNAs enriched with target genes in reciprocal direction. Only for those miRNAs found to be statistically significant, target genes were extracted which had their expression values in inverse direction with miRNA expression. miRNA-gene network was further created using miRNAs as source nodes and genes as Target nodes in Cytoscape [21].

### Biological annotation of genes

Biologically relevant pathways were identified using web-tools like EnrichR and GeneMANIA. Enrichr is an open source, freely available enrichment analysis web-tool [22, 23]. We used the tool for identification of pathways in GO and KEGG using our genes of interest as input. Independently, we also used GeneMANIA for identification of functions of our genes of interest. GeneMANIA is a user-friendly web interface used for predicting the function of genes using databases like GEO, BioGRID, Pathway Commons etc. [24, 25]. Network analysis of miR-gene interactions present in significantly enriched KEGG pathways were performed using miRNet.

### Identification of transcription factors

List of transcription factors were obtained from TcoF-DB v2 [26, 27] and compared with upregulated and downregulated genes to identify list of deregulated genes which could also act as transcription factors. The same was validated using other transcription factor databases like TRRUST v2 [28, 29] and TF2DNA [30], considering them only when they were present in at least two out of three databases.

## Results

### Description of selected datasets and overall plan

We have performed two meta-analysis in this study. The first one was using three miRNA microarray datasets to find out the miRNA meta-signature in serum of the PDAC patients as compared to normal individuals. The second one was using five miRNA microarray datasets to find out miRNA deregulation in PDAC tumor tissues as opposed to normal pancreas. Table 1 summarizes the description of these datasets. The datasets used in the first meta-analysis were PC_NAN_SG1 (our experimental result, Accession number: GSE140196), GSE59856 [31] and GSE85589 (unpublished result) complying with the selection criteria as mentioned in the ‘Methods’ section. First dataset (PC_NAN_SG1) was the result of our miRNA microarray experiment. We used samples from 2 PDAC and 2 normal individuals for the study to look at the differential expression of the miRNAs present in their serum using Affymetrix Multispecies miRNA-4.0 Array. The second dataset (GSE59856) compared serum miRNA expression levels in 100 pancreatic cancer patients and 150 healthy normals using 3D-Gene Human miRNA platform while the third dataset (GSE85589) evaluated the serum miRNA expression profile in 80 pancreatic cancer patients and 18 healthy normal individuals using the same array platform as ours.

**Table 1:** Information on datasets used in this study

Sl no	Dataset ID	Sample type	No. of Samples available	No. of Samples used	Platform	Normalization Method	Published literature
1	PC_NAN_SG1 (GSE140196)	Serum	2 PC, 4 N	2 PC, 2 N	[miRNA-4] Affymetrix Multispecies miRNA-4 Array	RMA-quantile	–
2	GSE59856	Serum	103 PC, 162 N	100 PC, 150 N	3D-Gene Human miRNA V20_1.0.0	Quantile	Kojima M et al., 2015 PMID:25706130
3	GSE85589	Serum	88 PC, 19 N	80 PC, 18 N	[miRNA-4] Affymetrix Multispecies miRNA-4 Array	RMA-quantile	–
4	GSE24279	Tissue	136 PC, 22 N	136 PC, 22 N	Febit human miRBase v11	VSN	Bauer AS et al.,2012 PMID:22511932
5	GSE32678	Tissue	25 PC, 7 N	18 PC, 4 N	miRCURY LNA microRNA Array, v.11.0, multispecies	VSN	Donahue TR et al.,2012 PMID:22261810
6	GSE41369	Tissue	9 PC, 9 N	9 PC, 7 N	NanoString nCounter Human miRNA assay (v1)	Quantile	Frampton AE et al.,2104 PMID:24120476
7	GSE43796	Tissue	6 PC, 5 N	6 PC, 3 N	Agilent-031181 Unrestricted_Human_miRNA_V16.0	Quantile	Park M et al.,2014 PMID:24072181
8	E-MTAB-753	Tissue	17PC,17 N	14PC,11 N	Affymetrix GeneChip miRNA 2.0 Array [miRNA-2_0]	RMA-quantile	Piepoli A et al.,2012 PMID:22479426

On the other hand, five datasets were selected following the inclusion and exclusion criteria for meta-analysis of miRNAs deregulated in pancreatic tumor tissues. GSE24279 was the first dataset comparing miRNA expression profile from 136 pancreatic tumor tissues and 22 normal pancreas using array platform from Febit [32]. While the second dataset, GSE32678, compared the same from 18 pancreatic cancer patients and 4 normal individuals using miRCURY LNA array [33], the third dataset (GSE41369) used Nanostring nCounter array platform with samples from 9 pancreatic cancer patients and 7 normal individuals [34]. The last two datasets (GSE43796 and E-MTAB-753) used Agilent and Affymetrix GeneChip miRNA array respectively with sample sizes of 6 Pancreatic cancer/ 3 normal and 17 pancreatic cancer/ 17 normal respectively [35, 36].

The overall plan of the study has been described in Fig. 1, which showed that the top ranked miRNAs from both the meta-analysis were compared to get the list of common miRNAs which might be representative of the fraction of miRNAs deregulated in pancreatic tumors and secreted in the circulation. Furthermore, target mRNAs of these miRNAs were obtained and a miR-gene interaction list was derived based on their expression in pancreatic tumors. Subsequently, using this miR-gene interaction information, network analysis was performed and perturbed biological pathways were identified.

### Cluster analysis of the datasets

**Fig. 2:**
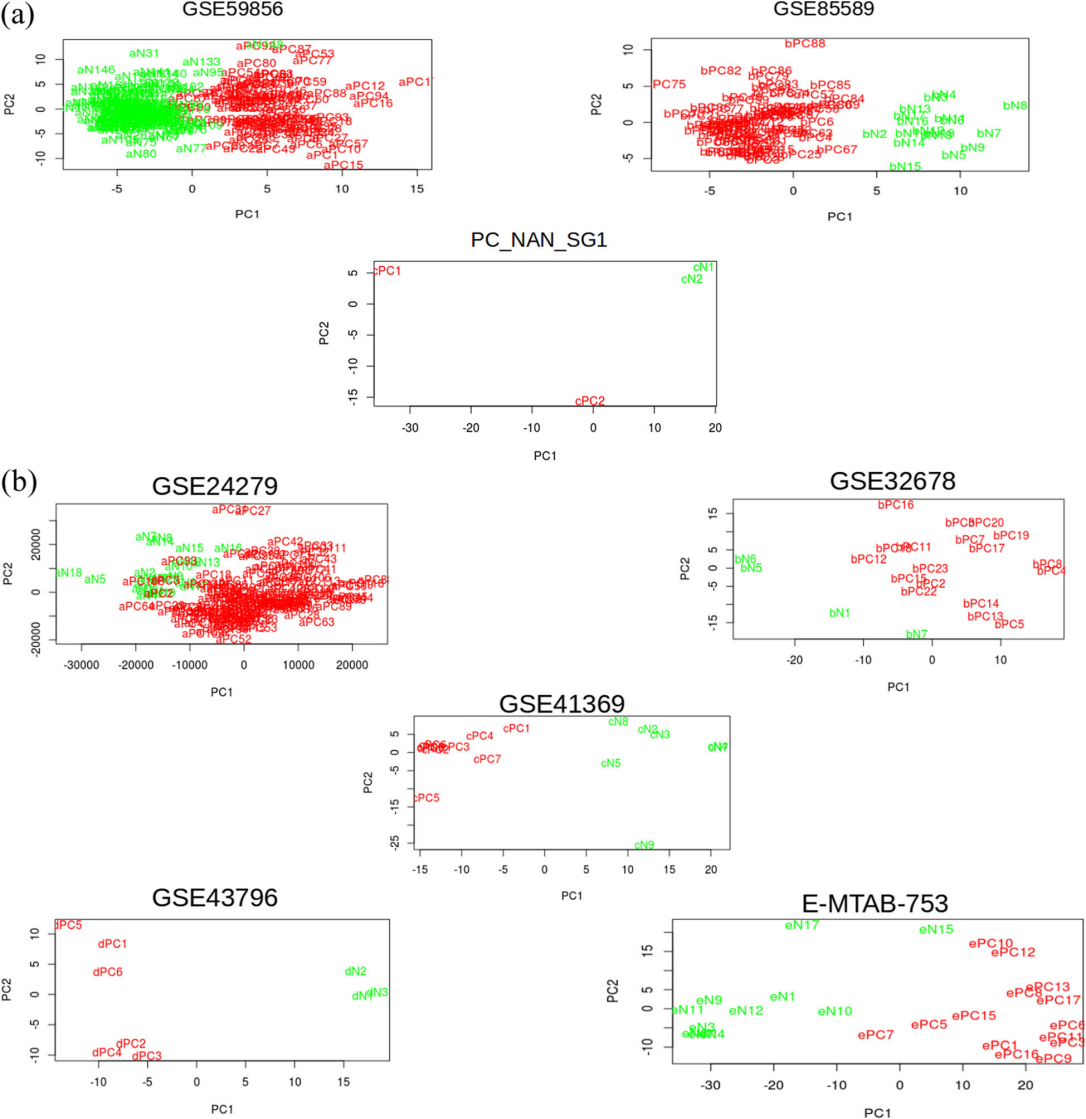
Principle Component Analysis of case and control samples from the datasets used for meta-analysis. **a** Three datasets with serum miRNA expression profiling and **b** Five datasets with tissue miRNA expression profiling

Unsupervised hierarchical cluster analysis was performed for all the datasets used for serum and tissue miRNA analysis. Principle component analysis (PCA) was also performed followed by removal of the outlier samples in these datasets in order to minimize non-specific effects as much as possible while processing these samples further. Figure 2a shows the PCA analysis of serum microarray datasets while Fig. 2b shows the same results for tissue miRNA datasets. We find in both the panels corresponding to serum and tissue, the individuals belonging to normal and pancreatic cancer fall in independent clusters.

### miRNA meta-signature of pancreatic ductal adenocarcinoma

**Fig. 3:**
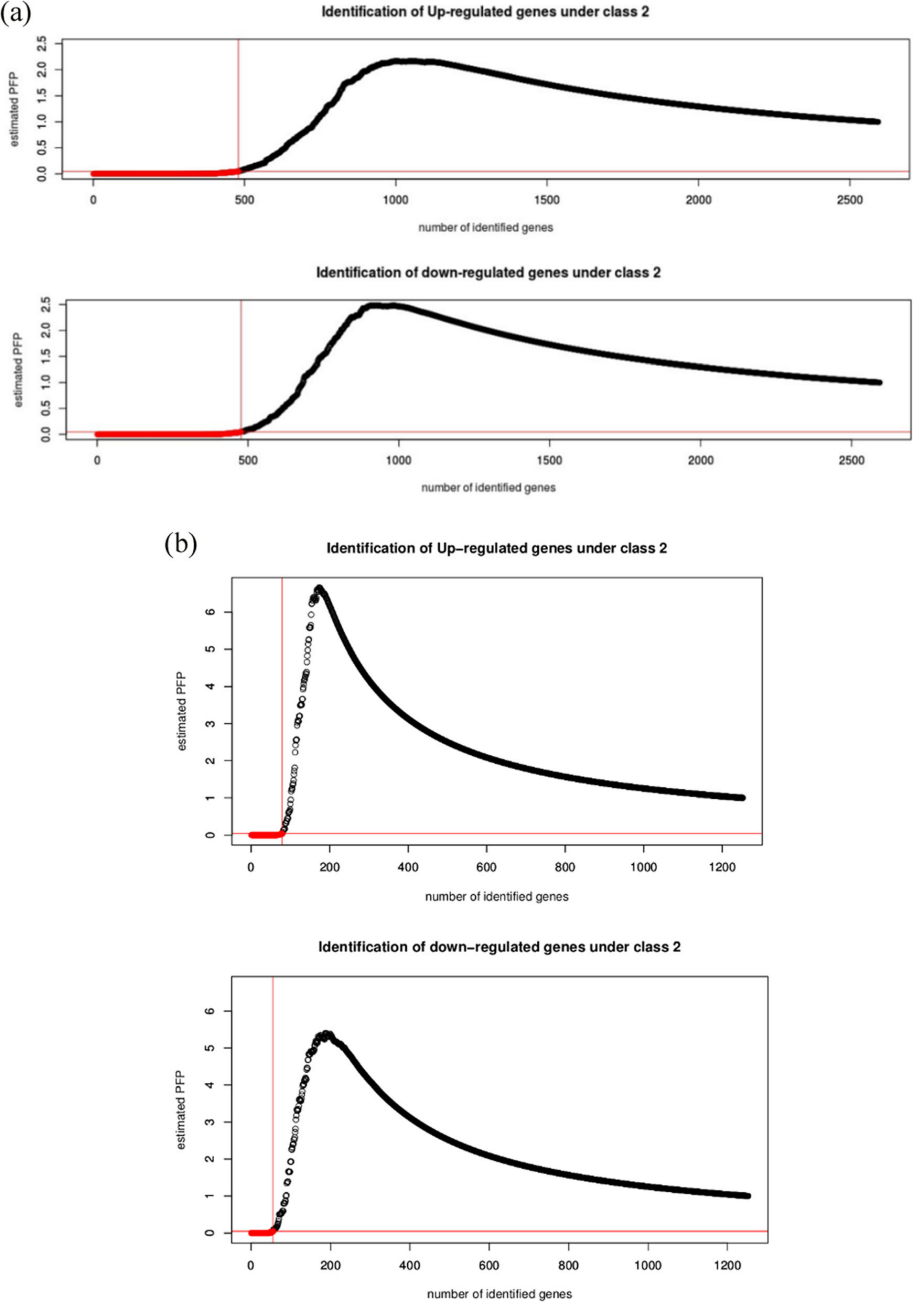
Selection of miRNAs within PFP cut-off of 0.05. Number of genes (miRNAs) in x-axis is plotted against estimated PFP (percentage of false prediction) in y-axis. **a** shows results from serum and **b** shows results from tissue. Red colour in figure represents genes falling within PFP cut-off of 0.05

Using RankProduct method, differentially expressed miRNAs were identified. DEmiRs were selected based on their percentage of false positives or PFP (with a cut-off of PFP < 0.05) and thereby, we obtained a meta-signature of miRNAs in pancreatic cancer. This meta-signature has two components as we have performed two different meta-analysis. Our analysis of expression profile of serum miRNAs differentially expressed in PDAC patients as obtained from three datasets resulted in 406 upregulated and 401 downregulated miRNAs. The result is shown in Fig. 3a, where number of miRNA identified is shown in red when number of genes (miRNAs) was plotted against the estimated PFP. Similarly, meta-analysis of 5 datasets with tissue miRNA profiling, yielded 77 upregulated and 53 downregulated miRNAs as shown in Fig. 3b. We also provide with a list of all differentially expressed miRNAs (both upregulated and downregulated) in serum and tissues, which have been shown in Additional file 2: Table S1a, b and Additional file 3: Table S2a and b respectively. The analysis package itself takes care of the heterogeneity between the samples.

### Identification of miRNAs secreted in serum of PDAC patients

A subset of the serum miRNAs, found to be characteristically altered in PDAC patients, must be an outcome of direct contribution from pancreatic tumor itself, either in the form of exosomal secretion or resulting from lysed tumour cells. We were interested to identify this fraction of the miRNAs in order to have a true picture of tumour derived miRNAs in serum. Our two meta-analysis yielded two different lists of differentially expressed miRNAs in serum and pancreatic tumour tissue of PDAC patients. We separately compared the up and downregulated miRNAs between these lists. This comparison ultimately resulted in 17 upregulated and 14 downregulated miRNAs common between them and hence believed to be the reflection of their expression changes in tissue into the serum of the patients (Table 2). We further undertook an extensive literature search in order to find evidences of these miRNAs to be involved in pancreatic cancer [36–70]. While we found many of them having their involvement in PDAC already reported, we also found miRNAs like let7f-5p, miR-1260b, miR-1914-3p, miR-30e-5p, miR-3137 and miR-3652 detected to be deregulated in PDAC for the first time. Moreover, we were interested to find out how many of these secretory miRNAs were part of exosomal cargo and comparison with the reported list of miRNAs from ExoCarta and MiRandola demonstrated that, apart from miR-3162-5p, all the 30 miRNAs were detectable as exosomal cargo in serum in different experimental set ups. Hereby, we generate a list of upregulated and downregulated miRNAs altered in pancreatic tumor tissue and most likely secreted into the serum of the patients through exosomes.

**Table 2:** Information on miRNAs, differentially expressed in both serum and tissues, as found in this study

Sl. no	miRNA	Regulation	Exosomally secreted	References	PMID
1	hsa-miR-103a-3p	up	Yes	Piepoli et al.2012 [36]	22479426
				Zhou H et al.2014 [37]	24984703
2	hsa-miR-1246	up	Yes	Piepoli et al.2012 [36]	22479426
				Ali et al.2012 [38]	22929886
				Hasegawa S et al.2014 [39]	25117811
3	hsa-miR-191-5p	up	Yes	Piepoli et al.2012 [36]	22479426
				Kent OA et al.2009 [40]	20037478
				Nakata et al.2011 [41]	22018284
				Jamieson NB et al.2012 [42]	22114136
				Liu H et al.2014 [43]	25168367
4	hsa-miR-210-3p	up	Yes	Nakata et al.2011 [41]	22018284
				Schultz et al.2012 [44]	22878649
				Papaconstantinou et al.2013 [45]	22850622
				Wang J et al.2009 [46]	19723895
				Ho AS et al.2010 [47]	20360935
				Chen WY et al.2012 [48]	22672828
				Takikawa T et al.2013 [49]	23831622
5	hsa-miR-23a-3p	up	Yes	Piepoli et al.2012 [36]	22479426
				Jamieson NB et al.2012 [42]	22114136
6	hsa-miR-320a	up	Yes	Nakata et al.2011 [41]	22018284
				Ali et al.2012 [38]	22929886
				Piepoli et al.2012 [36]	22479426
				Xin L et al.2017 [50]	28074846
				Wang W et al.2016 [51]	27279541
7	hsa-miR-320b	up	Yes	Xin L et al.2017 [50]	28074846
8	hsa-miR-320c	up	Yes	Xin L et al.2017 [50]	28074846
9	hsa-miR-320d	up	Yes	Xin L et al.2017 [50]	28074846
10	hsa-miR-331-3p	up	Yes	Nakata et al.2011 [41]	22018284
				Piepoli et al.2012 [36]	22479426
11	hsa-miR-423-3p	up	Yes	Nakata et al.2011 [41]	22018284
12	hsa-miR-4306	up	Yes	Madhavan B et al.2015 [52]	25388097
				Huang J et al.2016 [53]	27795830
13	hsa-miR-4317	up	Yes	NA	NA
14	hsa-miR-652-3p	up	Yes	Nakata et al.2011 [41]	22018284
15	hsa-miR-92a-3p	up	Yes	Piepoli et al.2012 [36]	22479426
				Ohuchida et al.2012 [54]	22407312
16	hsa-miR-92b-3p	up	Yes	Long M et al.2017 [55]	29078789
17	hsa-miR-99a-5p	up	Yes	Nakata et al.2011 [41]	22018284
				Nagao et al.2012 [56]	21983937
18	hsa-let-7f-5p	Down	Yes	NA	NA
19	hsa-miR-126-3p	Down	Yes	Nakata et al.2011 [41]	22018284
				Piepoli et al.2012 [36]	22479426
				Hamada et al.2011 [57]	22064652
				Zhou X et al.2016 [58]	27626307
				Feng SD et al.2017 [59]	29200874
				Frampton AE et al.2012 [60]	22845403
20	hsa-miR-1260b	Down	Yes	NA	NA
21	hsa-miR-16-5p	Down	Yes	Ohuchida et al.2012 [54]	22407312
				Jamieson NB et al.2012 [42]	22114136
				Kent OA et al.2009 [40]	20037478
				Gao L et al.2014 [61]	24600978
				Basu A et al.2010 [62]	22966344
				Li Y et al.2016 [63]	26929739
22	hsa-miR-1914-3p	Down	Yes	NA	NA
23	hsa-miR-26a-5p	Down	Yes	Ali et al.2012 [38]	22929886
				Laurila et al.2012 [64]	22344632
				Deng J et al.2013 [65]	24116110
				Fu X et al.2013 [66]	24114270
				Fukumoto I et al.2016 [67]	26490187
24	hsa-miR-26b-5p	Down	Yes	Kent OA et al.2009 [40]	20037478
				Nakata et al.2011 [41]	22018284
				Kaur S et al.2015 [68]	26605323
25	hsa-miR-30a-5p	Down	Yes	Jamieson NB et al.2012 [42]	22114136
				Yang C et al.2017 [69]	29052509
26	hsa-miR-30b-5p	Down	Yes	Nakata et al.2011 [41]	22018284
27	hsa-miR-30d-5p	Down	Yes	Jamieson NB et al.2012 [42]	22114136
28	hsa-miR-30e-5p	Down	Yes	NA	NA
29	hsa-miR-3137	Down	Yes	NA	NA
30	hsa-miR-3162-5p	Down	No	Lin MS et.2014 [70]	25664025
31	hsa-miR-3652	Down	Yes	NA	NA

### Identification of validated target genes of selected miRNAs

The altered set of miRNAs in PDAC, as obtained from our combined meta-analysis, must be performing important functions in the development and progression of the disease. The first step to elucidate their role is to identify the genes being targeted by these miRNAs. We preferred to focus only on experimentally validated targets and chose the web-tool miRNet which provides experimentally validated target information derived from multiple methods from high throughput experiments like CLASH, PAR-CLIP, Microarray and also from qPCR and reporter assays. We found validated target information for 21 out of 31 deregulated miRNAs. A total of 5935 validated targets (2920 genes targeted by 11 upregulated miRNAs and 4340 genes targeted by 10 downregulated miRNAs) were identified. Entire list of upregulated and downregulated target genes have been shown in Additional file 4: Table S3a and b respectively. Thus, we obtained a list of experimentally validated targets for 21 deregulated miRNAs specific for PDAC to be explored further for their involvement in the disease.

### Selection of miR-gene pairs

**Fig. 4:**
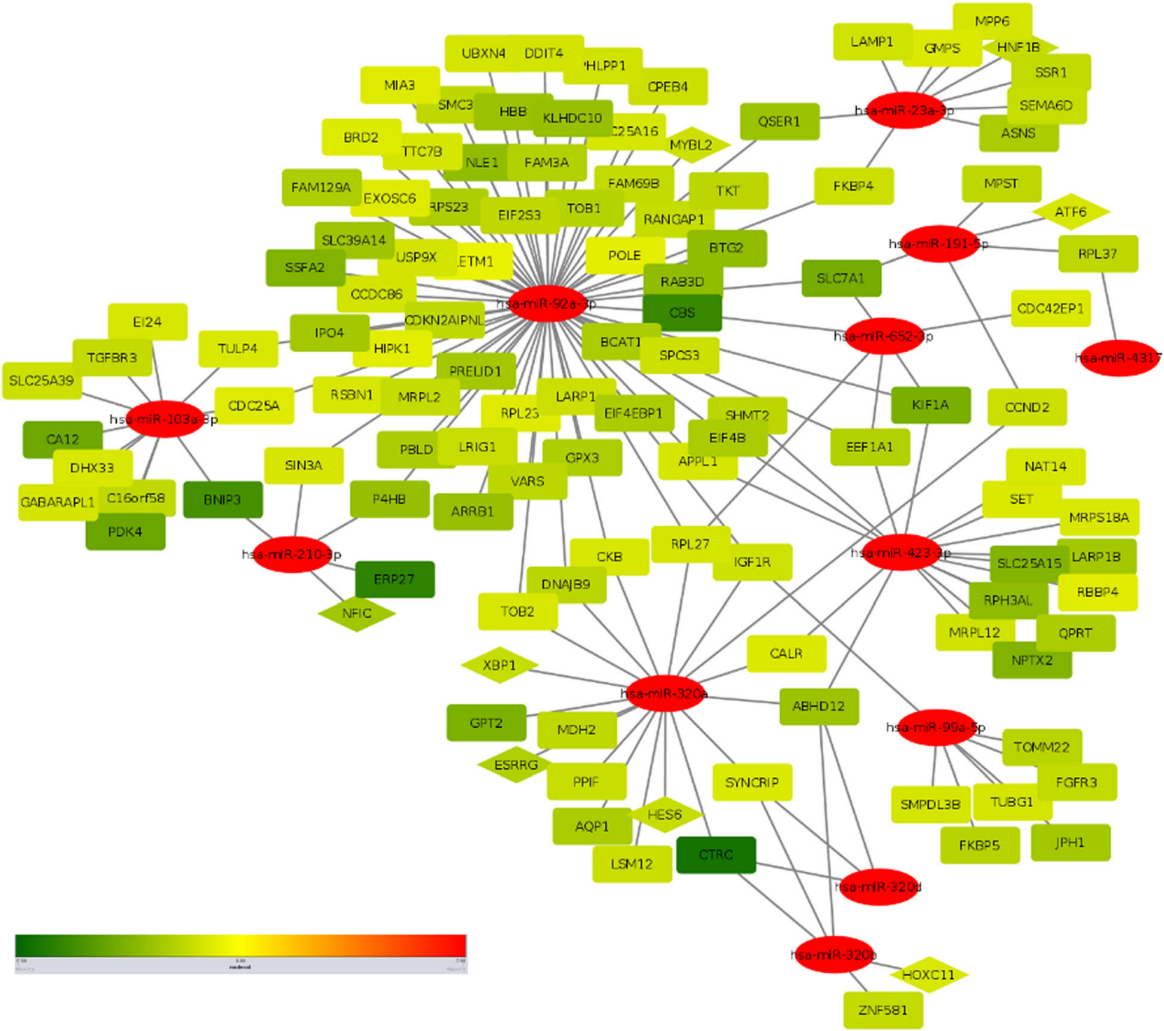
Interaction network between downregulated miRNAs and their target genes. miR-gene interaction network with downregulated miRNAs and their upregulated target genes. Colour scale is in increasing order of LFC from green to red i.e. green is downregulated and red is upregulated. Oval shape represents miRNA, rectangle represents target genes and triangles represent transcription factors which are being targeted by miRNAs

**Fig. 5:**
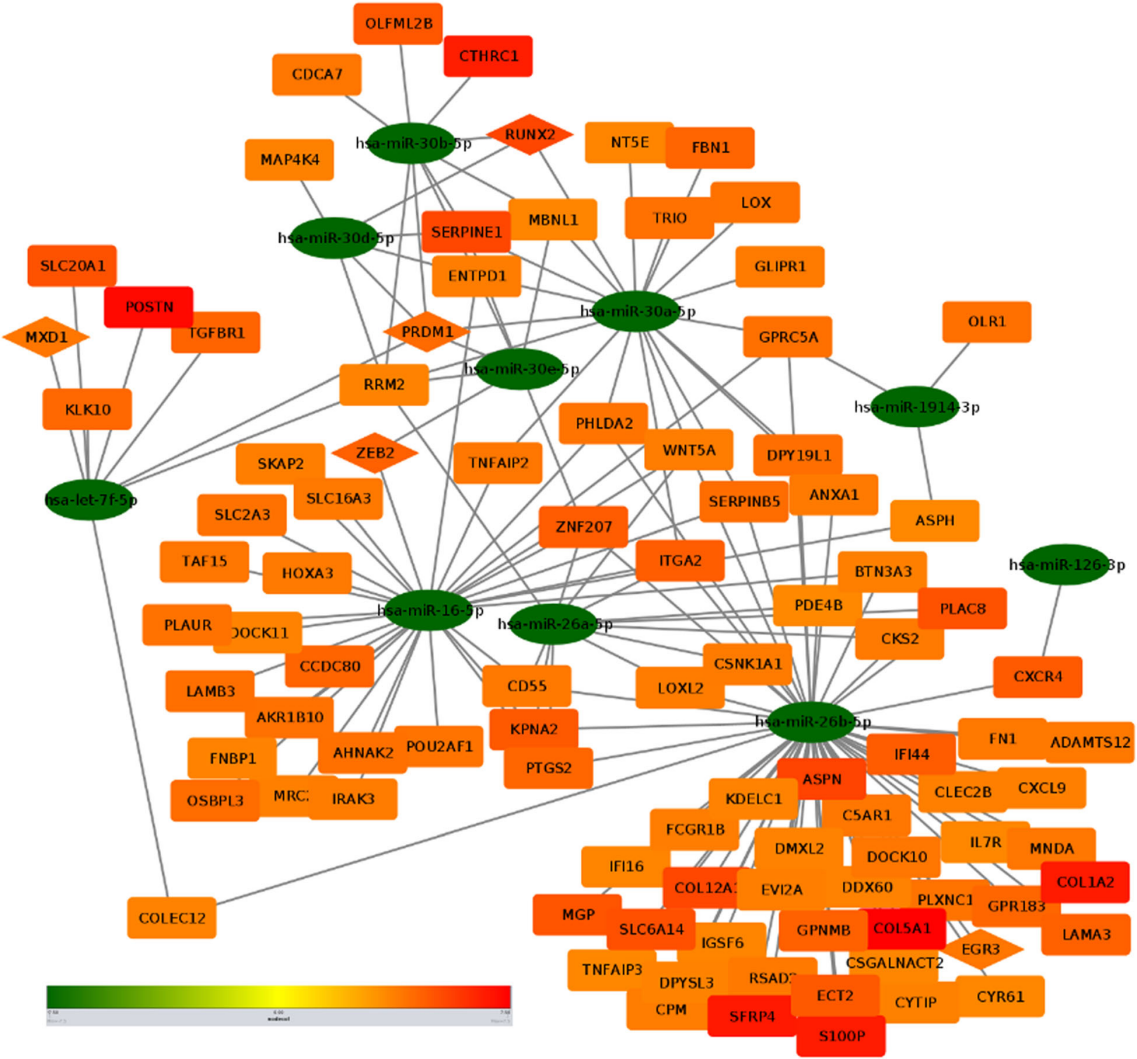
Interaction network between upregulated miRNAs and their target genes. miR-gene interaction network with upregulated miRNAs and downregulated target genes. Colour scale is in increasing order of LFC from green to red i.e. green is downregulated and red is upregulated. Oval shape represents miRNA, rectangle represents target genes and triangles represent transcription factors which are being targeted by miRNAs

The list of experimentally validated target genes was further investigated for their deregulation in PDAC. It is imperative that there is a huge tissue and disease specific differences in the miRNA regulation of gene expression. So, all these target genes of the altered miRNAs are definitely not involved in PDAC. Furthermore, even if we identify a target gene whose expression is being altered in pancreatic cancer, it might not be an actual target of that particular miRNA if we do not find an inverse correlation between their expressions. Hence, we explored pancreatic expression database (PED) to select that specific subset of target genes which are reported to be upregulated or downregulated in PDAC and whose expression is inversely correlated with that of the miRNAs. A hypergeometric test was conducted and all 21 miRNAs found to be statistically significant (Table 3). Next, target genes for those miRNAs were extracted having expression values in inverse direction with miRNA expression. We obtained 1149 such miRNA-gene pairs following these criteria. Top 300 (150 upregulated miR - downregulated target, 150 downregulated miR - upregulated target) interactions were further used to construct a miR-gene interaction network in Cytoscape to have a holistic view of how there is a concerted interaction of different miRNAs targeting the important genes relevant for PDAC (Figs. 4 and 5). All the miR-gene interaction pairs could be found in Additional file 5: Table S4.

**Table 3:** Results from Hypergeometric test used to find miRNAs enriched with Target genes in inverse direction of expression

Sl.no	miRNA	*P* value	*P* adjusted
1	hsa.let.7f.5p	4.87E-103	1.34E-102
2	**hsa.miR.103a.3p**	3.59E-19	5.64E-19
3	hsa.miR.126.3p	1.90E-34	3.48E-34
4	hsa.miR.16.5p	3.16E-285	3.48E-284
5	**hsa.miR.191.5p**	7.31E-13	8.93E-13
6	hsa.miR.1914.3p	1.71E-48	3.77E-48
7	hsa.miR.210.3p	7.35E-11	8.08E-11
8	**hsa.miR.23a.3p**	1.47E-17	2.15E-17
9	**hsa.miR.26a.5p**	6.19E-165	3.40E-164
10	hsa.miR.26b.5p	0.00E+ 00	0.00E+ 00
11	hsa.miR.30a.5p	8.13E-209	5.96E-208
12	hsa.miR.30b.5p	3.72E-157	1.63E-156
13	hsa.miR.30d.5p	5.17E-118	1.90E-117
14	hsa.miR.30e.5p	3.15E-117	9.89E-117
15	**hsa.miR.320a**	2.42E-31	4.09E-31
16	**hsa.miR.320b**	1.59E-12	1.85E-12
17	**hsa.miR.320d**	2.22E-08	2.33E-08
18	**hsa.miR.423.3p**	6.55E-37	1.31E-36
19	**hsa.miR.4317**	2.51E-03	2.51E-03
20	**hsa.miR.652.3p**	1.22E-13	1.58E-13
21	**hsa.miR.92a.3p**	2.60E-79	6.37E-79

Upregulated miRNAs are shown in bold, while downregulated miRNAs are shown in normal font

### Analysis of biological processes and pathways

**Fig. 6:**
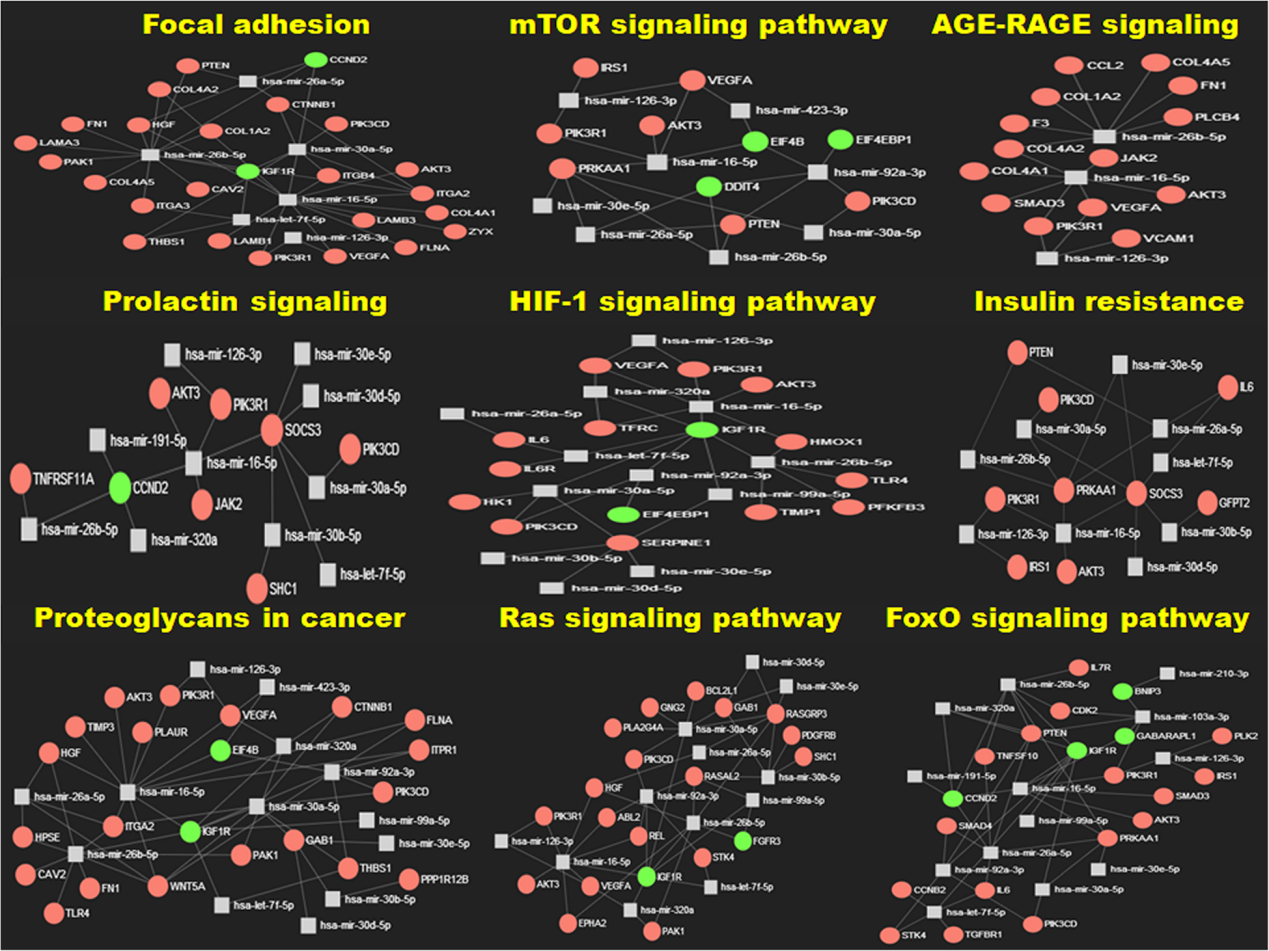
Sub-networks showing miRNA-gene interactions. Sub-networks depicting miRNA-gene interactions encompassing some of the significantly enriched KEGG pathways; red circle denotes up-regulated genes; green circles denote down-regulated genes and squares represent miRNA

Genes do not function in isolation, rather selected gene products cross-talk between each other being part of a pathway regulating specific biological processes. Hence, to have a comprehensive understanding of the role of the deregulated genes in the disease pathophysiology, we need to study them together to know how their alteration could perturb these pathways. We used multiple web-tools for this analysis and one of them, Enrichr, even performs the enrichment analysis based on which significantly associated pathways were selected for gene ontology: biological processes and KEGG pathways (adjusted *p*-value < 0.05). GeneMANIA is another web-tool which similarly predicts the functions of the genes and used for analogous purpose. Tables 4, 5 and 6 shows the representative pathways for biological processes (GO), pathways (KEGG) and functions (GeneMANIA) respectively along with the genes involved in them. The complete list of gene annotation could be found in Additional file 6: Table S5a, b and c. Comparison of the pathways or biological processes among these three categories clearly highlights some important aspects. Extracellular matrix (ECM) emerges as very important component and alteration in pathways like ECM-receptor interaction, focal adhesion and proteoglycan composition indicate importance of tumour-stroma interaction. The other cluster was the classical signaling pathways like TP53, HIF-1, PI3K-Akt, Jak-STAT, FoxO, mTOR, TGF-beta, NF-kB etc. which are altered in most cancers. We also found alteration in AGE-RAGE signaling pathway, insulin resistance signaling pathway and prolactin signaling pathway, which were less discussed in pancreatic cancer. Interestingly, there were few pancreas specific pathways where some of them were clearly indicative of chronic inflammatory process developing into malignancy. We further performed miR-gene network analysis using miRNet and also selected most important pathways as appeared in KEGG, shown in Fig. 6. Main network from which the sub-networks were extracted could be found in Additional file 1: Figure S1.

**Table 4:** List of 20 significant ‘GO Biological process’ that were obtained using miRNA-targeted genes in Pancreatic Cancer

Sl.no	Term	Adjusted *P*-value	Genes
1	PI3K-Akt signaling pathway_Homo sapiens_hsa04151	2.96E-09	*PRKAA1;PHLPP1;IRS1;ITGB4;LAMA3;PTEN;PIK3CD;PIK3R1;THBS1;IGF1R;GNG2;CCND2;AKT3;EIF4EBP1;JAK2;IL6R;EIF4B;MCL1;PDGFRB;IFNAR2;LAMB3;ITGA3;HGF;ITGA2;FN1;LAMB1;PPP2R5C;VEGFA;IL6;COL1A2;CREB1;COL4A2;CCNE2;COL4A1;CDK2;DDIT4;COL4A5;IL7R;TLR4;FGFR3;EPHA2;BCL2L1*
2	AGE-RAGE signaling pathway in diabetic complications_Homo sapiens_hsa04933	4.42E-08	*SMAD4;VCAM1;SMAD3;SERPINE1;FN1;PIK3CD;PIK3R1;F3;TGFBR1;VEGFA;IL6;COL1A2;PLCB4;COL4A2;COL4A1;AKT3;BAX;COL4A5;CCL2;JAK2*
3	Focal adhesion_Homo sapiens_hsa04510	9.82E-08	*SHC1;ITGB4;LAMA3;PTEN;PIK3CD;PIK3R1;THBS1;IGF1R;PAK1;CCND2;AKT3;FLNA;PDGFRB;LAMB3;CAV2;ITGA3;HGF;ITGA2;FN1;LAMB1;VEGFA;COL1A2;COL4A2;COL4A1;ZYX;COL4A5;CTNNB1;PPP1R12B*
4	p53 signaling pathway_Homo sapiens_hsa04115	1.09E-06	*RRM2;EI24;SERPINE1;PTEN;THBS1;SERPINB5;CCNB2;TP53I3;CCND2;CCNE2;CDK2;CHEK1;BAX;PMAIP1;BID*
5	FoxO signaling pathway_Homo sapiens_hsa04068	3.69E-06	*PRKAA1;SMAD4;GABARAPL1;SMAD3;IRS1;PLK2;BNIP3;PTEN;PIK3CD;PIK3R1;STK4;TGFBR1;IGF1R;CCNB2;IL6;CCND2;AKT3;CDK2;TNFSF10;IL7R*
6	MicroRNAs in cancer_Homo sapiens_hsa05206	2.19E-05	*DNMT1;SLC45A3;BMPR2;IRS1;SHC1;PTEN;PTGS2;SLC7A1;THBS1;CCND2;STMN1;HMOX1;TIMP3;E2F3;MCL1;PDGFRB;KIF23;SERPINB5;CDC25A;VEGFA;CDC25B;ZEB2;CCNE2;DDIT4;FSCN1;VIM;ZFPM2;FGFR3;RECK;EZH2*
7	HIF-1 signaling pathway_Homo sapiens_hsa04066	1.21E-04	*PFKFB3;TFRC;SERPINE1;PIK3CD;PIK3R1;IGF1R;VEGFA;HK1;IL6;AKT3;EIF4EBP1;HMOX1;TIMP1;IL6R;TLR4*
8	Proteoglycans in cancer_Homo sapiens_hsa05205	1.37E-04	*CAV2;HGF;ITGA2;WNT5A;FN1;GAB1;ITPR1;PLAUR;PIK3CD;PIK3R1;THBS1;VEGFA;IGF1R;PAK1;AKT3;TIMP3;CTNNB1;FLNA;HPSE;PPP1R12B;TLR4;EIF4B*
9	ECM-receptor interaction_Homo sapiens_hsa04512	7.42E-04	*COL1A2;LAMB3;COL4A2;ITGA3;COL4A1;ITGB4;ITGA2;LAMA3;FN1;COL4A5;LAMB1;THBS1*
10	mTOR signaling pathway_Homo sapiens_hsa04150	9.57E-04	*PRKAA1;IRS1;AKT3;DDIT4;EIF4EBP1;PTEN;PIK3CD;PIK3R1;EIF4B;VEGFA*
11	Ras signaling pathway_Homo sapiens_hsa04014	1.58E-03	*PDGFRB;RAB5C;SHC1;HGF;GAB1;PLA2G4A;PIK3CD;PIK3R1;RASAL2;STK4;VEGFA;RASGRP3;IGF1R;PAK1;GNG2;AKT3;REL;ABL2;FGFR3;EPHA2;BCL2L1*
12	Pancreatic cancer_Homo sapiens_hsa05212	1.81E-03	*SMAD4;SMAD3;AKT3;TGFA;PIK3CD;E2F3;PIK3R1;TGFBR1;VEGFA;BCL2L1*
13	TNF signaling pathway_Homo sapiens_hsa04668	2.26E-03	*VCAM1;JAG1;LIF;TNFAIP3;PIK3CD;PIK3R1;CFLAR;PTGS2;SOCS3;IL6;CREB1;AKT3;CCL2*
14	TGF-beta signaling pathway_Homo sapiens_hsa04350	9.04E-03	*SMAD1;SMAD4;BMPR2;SMAD3;SMURF2;ID1;THBS1;TGFBR1;ACVR2A;SMAD7*
15	NF-kappa B signaling pathway_Homo sapiens_hsa04064	1.60E-02	*LYN;VCAM1;BLNK;TNFAIP3;CFLAR;TNFRSF11A;PTGS2;TLR4;MALT1;BCL2L1*
16	Transcriptional misregulation in cancer_Homo sapiens_hsa05202	1.85E-02	*SMAD1;MEF2C;SLC45A3;TAF15;RUNX2;IGF1R;IL6;CCND2;NR4A3;WT1;SIN3A;REL;ERG;RUNX1T1;BCL2L1*
17	Chemokine signaling pathway_Homo sapiens_hsa04062	2.43E-02	*LYN;CXCL9;SHC1;PIK3CD;CXCR4;ARRB1;PIK3R1;ARRB2;CXCL13;PAK1;GNG2;PLCB4;AKT3;CCL2;JAK2*
18	Prolactin signaling pathway_Homo sapiens_hsa04917	2.59E-02	*SOCS3;CCND2;SHC1;AKT3;PIK3CD;TNFRSF11A;PIK3R1;JAK2*
19	Jak-STAT signaling pathway_Homo sapiens_hsa04630	3.02E-02	*IFNAR2;LIF;PIK3CD;PIK3R1;SOCS3;IL6;CCND2;AKT3;IL7R;JAK2;IL6R;BCL2L1;MCL1*
20	Insulin resistance_Homo sapiens_hsa04931	3.41E-02	*SOCS3;PRKAA1;IL6;CREB1;IRS1;GFPT2;AKT3;PTEN;PIK3CD;PIK3R1*

**Table 5:** List of 20 significant ‘KEGG pathways’ that were obtained using miRNA-targeted genes in Pancreatic Cancer

Sl.no	Term	Adjusted *P*-value	Genes
1	regulation of cell proliferation (GO:0042127)	3.99E-11	*BTG2;CXCL9;BMPR2;IRS1;TGFB1I1;KIF14;PTEN;ADARB1;PTPRK;CXCL13;PKD2;IGF1R;CCND2;NAMPT;TIMP1;JAK2;IL6R;PDGFRB;HGF;RPL23;AXIN2;EMP3;PPP2R5C;TGFBR1;CDC25B;KAT2B;SFRP4;BIRC5;SHC1;EIF5A2;NPR3;CDCA7;TGFA;TTK;TNFRSF11A;THBS1;TOB1;ZFP36L1;EFNB2;TBRG1;NUAK1;PAK1;GPNMB;RBBP4;FRZB;ZNF703;ABL2;HAS2;SSR1;LYN;TFAP2A;XBP1;JAG1;FN1;HMGA1;LIF;NAP1L1;KLF4;VEGFA;CUL4A;SKI;IL6;NR4A3;WT1;DLC1;ACER3;BHLHE40;CDK2;CTNNB1;CALR;FAM98A;FOLR2;IL7R;FGFR3*
2	regulation of cell migration (GO:0030334)	1.97E-08	*CLIC4;PLXND1;SERPINE2;ZMYND8;SERPINE1;KIF14;PTEN;PIK3CD;MIA3;PRKX;PRR5L;ADARB1;LDB2;PTPRK;RND3;THBS1;CYR61;RHOBTB1;CORO1C;IGF1R;DOCK10;PAK1;GPNMB;DPYSL3;ZNF703;FLNA;HAS2;PLXNA1;PLXNC1;PDGFRB;XBP1;ANXA1;JAG1;HGF;LAMB1;TGFBR1;SMAD7;VEGFA;DLC1;RECK*
3	extracellular matrix organization (GO:0030198)	2.89E-08	*ITGB4;COL12A1;ITGB2;LAMA3;SERPINE1;HTRA1;THBS1;LOXL2;SH3PXD2A;SERPINH1;HAS2;TIMP1;JAM2;POSTN;VCAM1;LAMB3;ITGA3;ITGA2;FN1;LAMB1;TGFBR1;COL1A2;COL4A2;COL5A1;LOX;COL4A1;COL4A5;COL8A1;LCP1;TGFBI;RECK;MATN3;FBN1*
4	positive regulation of transcription, DNA-templated (GO:0045893)	5.06E-06	*BMPR2;TGFB1I1;SERPINE1;ARRB1;AHR;IKZF1;LDB2;IKZF2;PKD2;HOXC11;LITAF;NAT14;CYR61;SIN3A;CHEK1;NAMPT;MYBL2;MEF2C;HGF;RPL23;ATRX;TCF12;WNT5A;HNF1B;RUNX2;TGFBR1;KAT2B;PLSCR1;ZEB2;CREB1;ELF4;MTF2;TET3;CKS2;ERG;ZFPM2;ATF6;HOXB7;TLR4;NFAT5;RNASEL;SHC1;PIK3R1;MRPL12;ARNTL2;DHX33;IFI16;E2F3;HIVEP3;ZNF423;MAP3K2;TFAP2A;SMAD1;SMAD4;XBP1;JAG1;SMAD3;TAF15;BCL11B;FOXF2;HMGA1;LIF;ESRRG;KLF4;TRERF1;ACVR2A;SMAD7;VEGFA;SKI;IL6;NR4A3;WT1;NFIC;POU2AF1;CAPRIN2;REL;CTNNB1;TCF4;SSBP2;PHF19*
5	cytokine-mediated signaling pathway (GO:0019221)	8.46E-05	*CXCL9;IRS1;ITGB2;PIK3CD;CXCL13;LMNB1;CFL1;TIMP1;JAK2;IL6R;TRIM22;IFNAR2;ANXA1;SERPINB2;RSAD2;HGF;IRAK3;ASPN;PSME4;FSCN1;BIRC5;SNRPA1;LCP1;RNASEL;SHC1;FPR1;PIK3R1;TNFRSF11A;PTGS2;TANK;SOCS3;INPP5D;CCL2;HMOX1;GBP2;FCGR1B;MCL1;SMAD4;VCAM1;FN1;LIF;PSMB8;VEGFA;IL6;COL1A2;P4HB;VIM;IL7R;BCL2L1;LIMS1*
6	transforming growth factor beta receptor signaling pathway (GO:0007179)	1.14E-04	*SMAD1;SMAD4;SMAD3;USP9X;ARRB2;PTPRK;TGFBR1;SKI;TGFBR3;COL1A2;ID1;ZYX;ADAM9;FERMT2*
7	regulation of epithelial to mesenchymal transition (GO:0010717)	1.63E-04	*SMAD4;SMAD3;TGFB1I1;PTEN;AXIN2;TGFBR1;LOXL2;PBLD;SMAD7;ZNF703;CTNNB1;PHLDB2;EZH2*
8	positive regulation of cell migration (GO:0030335)	1.63E-03	*PDGFRB;DOCK5;NRP1;XBP1;BMPR2;SMURF2;HGF;FN1;GAB1;PIK3CD;LAMB1;MIA3;THBS1;CYR61;RTN4;TGFBR1;IGF1R;VEGFA;PAK1;GPNMB;ZNF703;HAS2*
9	cell-matrix adhesion (GO:0007160)	4.62E-03	*VCAM1;ITGA3;ITGB4;DLC1;ITGA2;ITGB2;ZYX;ADAM9;HPSE;PTPRK;ADAMTS12;FERMT2*
10	regulation of ERK1 and ERK2 cascade (GO:0070372)	5.09E-03	*LYN;PDGFRB;EPHA4;NRP1;SMAD4;SHC1;C5AR1;FN1;PTEN;ARRB1;ARRB2;SYNJ2BP;CYR61;CTGF;IGF1R;GPNMB;GPR183;CCL2;TIMP3;FGFR3;TLR4;EPHA2*
11	positive regulation of leukocyte chemotaxis (GO:0002690)	1.08E-02	*CXCL9;IL6;SERPINE1;WNT5A;CALR;CXCL13;IL6R;THBS1;VEGFA*
12	transmembrane receptor protein tyrosine kinase signaling pathway (GO:0007169)	1.13E-02	*NRP1;BMPR2;IRS1;SHC1;PIK3R1;PIK3C2A;IGF1R;EFNB2;PAK1;SPRED1;STMN1;BLNK;PDK4;ABL2;JAK2;APPL1;LYN;PDGFRB;ACTR2;EPHA4;CAV2;HGF;GAB1;AP2B1;VEGFA;DDIT4;FGFR3;CDK5R1;EPHA2*
13	proteoglycan metabolic process (GO:0006029)	1.69E-02	*BMPR2;CSGALNACT2;FAM20B;HPSE;ADAMTS12*
14	insulin receptor signaling pathway (GO:0008286)	1.75E-02	*CAV2;IRS1;SHC1;GAB1;PDK4;PIK3R1;PIK3C2A;APPL1;IGF1R*
15	regulation of I-kappaB kinase/NF-kappaB signaling (GO:0043122)	2.73E-02	*SLC20A1;CARD8;PLK2;WNT5A;TNFAIP3;CFLAR;LITAF;TANK;MALT1;VAPA;REL;TNFSF10;FLNA;HMOX1;TRIM59;ECT2;TRIM22*
16	cellular response to reactive oxygen species (GO:0034614)	3.88E-02	*IL6;CDK2;PPIF;TNFAIP3;PTPRK;ECT2;PKD2;AQP1*
17	inflammatory response (GO:0006954)	3.97E-02	*LYN;CXCL9;ANXA1;C5AR1;ITGB2;FPR1;CXCR4;PIK3CD;TNFRSF11A;CXCL13;LYZ;THBS1;IL6;DLC1;BLNK;REL;CCL2;FOLR2;TLR4*
18	positive regulation of tumor necrosis factor biosynthetic process (GO:0042535)	4.39E-02	*TLR1;THBS1;TLR4*
19	protein sumoylation (GO:0016925)	4.69E-02	*IFIH1;ZNF451;PCNA;NUP155;NUP50;BIRC5;RANGAP1;RAE1*
20	cellular response to hypoxia (GO:0071456)	4.77E-02	*BNIP3;PSME4;UBE2D3;HMOX1;PMAIP1;P4HB;PTGS2;CPEB2;PSMB8;AQP1;VEGFA;ZFP36L1*

**Table 6:** List of 20 significant ‘GeneMANIA functions’ that were obtained using miRNA-targeted genes in Pancreatic Cancer

Sl.no	Function	FDR	Genes in network
1	protein kinase binding	4.84E-04	18
2	extracellular matrix organization	2.60E-03	17
3	angiogenesis	3.08E-03	15
4	cell cycle G2/M phase transition	3.08E-03	12
5	microtubule cytoskeleton organization	5.63E-03	14
6	nuclear division	6.74E-03	15
7	response to oxygen levels	8.77E-03	10
8	platelet-derived growth factor binding	8.79E-03	4
9	cell cycle checkpoint	1.11E-02	13
10	intrinsic apoptotic signaling pathway	1.13E-02	12
11	response to oxidative stress	1.31E-02	11
12	regulation of angiogenesis	1.40E-02	10
13	transmembrane receptor protein serine/threonine kinase signaling pathway	1.40E-02	13
14	integrin binding	1.42E-02	7
15	regulation of mitosis	3.14E-02	8
16	epithelial to mesenchymal transition	3.14E-02	7
17	regulation of release of cytochrome c from mitochondria	3.32E-02	5
18	cell chemotaxis	4.25E-02	10
19	endothelial cell proliferation	4.30E-02	7
20	Rho protein signal transduction	4.80E-02	6

## Transcription factors being targeted by DEmiRs

To have further insight into the functions of the miRNA target genes, we wanted to know how many of them are transcription factors (TFs) themselves. The purpose behind the approach was the fact that TFs are capable to alter an array of genes leading to a strong regulatory effect. Therefore, targeting a TF by a miRNA which is significantly altered in a disease should have much broader impact on the subsequent pathways as well as functioning of that cell. Hence, regulation of TF expression by miRNA plays a very important role in the disease process. We took help from different web-tools (as described in methods) to find out how many of the pancreatic cancer specific targets of that DEmiRs could also act as transcription factors. We identified 38 upregulated genes and 8 downregulated genes which are predicted to act as transcription factors from TcoF-DB v2, TRRUST v2 and TF2DNA shown in Tables 7 and 8. We also identified 15 upregulated genes and 5 downregulated genes acting as transcription cofactors from TcoF-DB v2 as shown in Additional file 7: Table S6.

**Table 7:** List of downregulated genes (targeted by upregulated miRNAs) which are reported to act as transcription factors as reported in at least two of the three TF databases i.e. TF/TcoF DB, TRRUST and TF2DNA

Sl.no	Gene	TF/TcoF DB	TRRUST	TF2DNA
1	HNF1B	YES	YES	NO
2	NFIC	YES	YES	YES
3	XBP1	YES	YES	YES
4	ATF6	NO	YES	YES
5	ESRRG	NO	YES	YES
6	HES6	NO	YES	YES
7	HOXC11	NO	YES	YES
8	MYBL2	NO	YES	YES

**Table 8:** List of upregulated genes (targeted by downregulated miRNAs) which are reported to act as transcription factors as reported in at least two of the three TF databases i.e. TF/TcoF DB, TRRUST and TF2DNA

Sl.no	TFS	TF/TcoF DB	TRRUST	TF2DNA
1	BNC2	YES	NO	YES
2	IKZF2	YES	NO	YES
3	RLF	YES	NO	YES
4	AHR	YES	YES	NO
5	CREB1	YES	YES	YES
6	CREM	YES	YES	YES
7	E2F3	YES	YES	NO
8	EGR3	YES	YES	YES
9	ELF4	YES	YES	YES
10	ERG	YES	YES	YES
11	EZH2	YES	YES	NO
12	FOXF2	YES	YES	YES
13	ID1	YES	YES	YES
14	IKZF1	YES	YES	YES
15	KLF4	YES	YES	YES
16	LCOR	YES	YES	NO
17	MEF2C	YES	YES	YES
18	NFAT5	YES	YES	YES
19	NR4A3	YES	YES	YES
20	PRDM1	YES	YES	YES
21	REL	YES	YES	YES
22	RUNX2	YES	YES	NO
23	SKI	YES	YES	NO
24	SKIL	YES	YES	NO
25	SMAD1	YES	YES	YES
26	SMAD3	YES	YES	NO
27	SMAD4	YES	YES	YES
28	TRPS1	YES	YES	YES
29	WT1	YES	YES	YES
30	ZEB2	YES	YES	YES
31	ARNTL2	NO	YES	YES
32	HOXB7	NO	YES	YES
33	MXD1	NO	YES	YES
34	SMAD7	NO	YES	YES
35	TCF12	NO	YES	YES
36	TCF4	NO	YES	YES
37	TFAP2A	NO	YES	YES
38	ZNF423	NO	YES	YES

## Discussion

Circulating miRNAs have been of much importance these days due to their potential to function as disease specific non-invasive biomarkers. It is not always necessary to know the origin or tissue-specificity of the specific miRNAs to designate them as biomarker for a specific disease. However, a subset of these circulating miRNAs in serum or plasma which is actually secreted from the diseased tissue or organ will provide additional information regarding regulation of gene expression taking place within that tissue, together with their role as biomarker. Unfortunately, not much attempts are there trying to combine these information in pancreatic ductal adenocarcinoma. There are many research papers, reviews and meta-analysis papers separately analyzing the altered miRNA profile in serum and tumour tissue of PDAC patients [5–7] but none of them have tried to compare themselves between them and individual studies also had lot of differences between their findings. While miR-21, miR-155, miR-1290, miR-210, miR-217, miR-141, miR-222, miR-196a, miR-494, miR-148b, miR-216, miR-375, miR-29c miR-96 etc. were among mostly deregulated in tumour tissues, miR-10b, miR18a, miR-20a, miR-21, miR-139-3p, miR-185, miR-210 and miR-196a were frequently altered in serum of the patients. In order to address this discrepancy, differentially expressed serum and tissue miRNAs between studies, we chose to focus on the fraction of serum miRNAs which is most likely secreted from the pancreatic tumour itself. Hence, the common miRNAs we find here has functional importance in the tissue as well as has potential to function as serum based biomarker for detection of PDAC.

The variation between the individual studies could primarily be attributable to the difference in sample sizes, sample processing methods, population differences, selection of platforms etc. The selection of normalization method was based on suggestions/ recommendations by the platform manufacturers and also based on the information on commonly used or preferred normalization methods by researchers using those platforms. Combining all the samples together and analyzing data from all of them following same statistical methodology would deliver results which should be more accurate than the individual analysis. The package, ‘Rankprod’ takes care of variation arise due to multiple studies addressing the clinical heterogeneity. Furthermore, identification of novel miRNAs deregulated in PDAC could be attributed to meta-analysis. It is the beauty of meta-analysis that it could detect the miRNAs which could not be detected in individual studies due to low sample size.

We preferred to select experimentally validated targets only and did not consider the miRNAs which did not have any experimentally validated targets for further analysis. Thereby, the number of miRNAs got reduced from 31 to 21 and Table 3 shows a list of these 21 miRNAs which can be designated as the ‘meta-signature’ of miRNAs for pancreatic cancer detectable in both serum and tumour tissues. Apart from miR-4317, all of the up-regulated miRNAs selected in our meta-analysis have already been reported to be overexpressed in PDAC. While miR-103a-3p has been shown to act as ‘driver’ of pancreatic cancer, the exact role of miR-191-5p, miR-210-3p, miR-23a-3p, miR-103a-3p, miR-92a and miR-320a/b has also been functionally characterized at the molecular level. It has been shown that miR-210-3p regulated the interaction between pancreatic stellate cells and pancreatic cancer cells, a phenomenon being very important with respect to development of PDAC. miR-320a has been shown to promote 5-FU resistance to pancreatic cancer cells by targeting PDCD4 and most interestingly, miR-320b overexpression have been correlated to late stage chronic pancreatitis, linking this chronic inflammatory disease of pancreas to PDAC. Similarly miR-23a-3p and miR-103a-3p have also been shown to target Epithelial Splicing Regulatory Protein 1 and oncogene GPRC5A respectively. miR-99a-5p has been found to be overexpressed in chemotherapy resistant PDAC [71] and though miR-652-3p was overexpressed and responsible for proliferation and metastasis in other cancers [72], a report showed its downregulation in PDAC [73]. Therefore, our identification of overexpressed miR-652-3p demands more experimental insight into the mechanism. miR-92a-3p is a known oncogene in other cancer and its role in PDAC has only been shown in pancreatic cancer cells where it targets JNK signaling pathway inhibitor, DUSP10 and promote JNK signaling and tumorigenesis. However, we show here upregulation of miR-92a in PDAC patients for first time. Following the same trend, there are no previous reports of involvement of miR-423-3p in PDAC and it is imperative that the role of miR-4317, miR-423-3p and miR-92a-3p in PDAC should also be explored in further details.

On the other hand, we also observed down-regulation of known tumour suppressor miRNAs in pancreatic cancer like let-7f-5p, miR-16-5p, miR-126-3p, miR-30d-5p. Functional characterization and how the down-regulation of miRNAs could affect tumourigenesis in pancreas has already been worked out for many of these repressed miRNAs as we find miR-126-3p targeting ADAM9, KRAS and CRK oncogene, while miR-26a suppressing cycling E2 mediated progression of cell cycle in pancreatic cancer. Furthermore we also observed reduced expression of three other miRNAs which are known to function as tumour suppressor miRNAs in other cancers [74–78], but there was one report for each of the miRNAs mentioning about their up-regulation in pancreatic cancer tissues or cell lines requiring additional exploration. Interestingly, we further report for the first time that miR-30b-5p and miR-30a-5p, which are known tumour suppressors in other cancers, are also down-regulated in pancreatic cancers, as observed from our findings.

Moreover, it was of concern that all the targets of one particular miRNA might not actually get targeted in PDAC. Hence, important aspect was to identify the target genes of these 21 miRNAs which actually are deregulated in PDAC in the inversely correlated direction with miRNAs. Pancreatic Expression Database was our choice and after comparing the results with the database entries, we constructed a miRNA-gene interaction table recording all possible interaction of DEmiRs with their DE-Targets in PDAC (Additional file 5: Table S4). However, identifying the miRNA-target interaction alone doesn’t explain the biology completely. The most important part was to identify the key pathways where those genes contributed and deregulation of those genes resulted in perturbation of the pathways. We used EnrichR and GeneMANIA and got almost similar pathways altered and enriched in both cases. Among them, pathways like AGE-RAGE pathways, prolactin signaling pathway and insulin resistance signaling pathway were of much interest as they were not explored in PDAC in much detail. Advanced glycation end products (AGE) are complex group of compounds and interaction of AGEs with their specific receptors (RAGEs) has important functional implications. RAGE has been found to be overexpressed in multiple cancers like colorectal, lung, oral, brain, prostate, melanoma, lymphomas and ovarian cancers [79]. RAGE is known as a multi-ligand receptor, as not only AGE but several other ligands like, HMGB1, and S-100 (calgranulins) etc. also bind to RAGE. RAGE activation is known to promote tumor vasculature, tumor growth and invasion through alteration of various pathways [80]. RAGE signaling has also been implicated earlier in pancreatic cancer development where loss of RAGE function inhibited the development of PDAC in mouse models [81–83]. The immuno-histochemical analysis confirmed the expression of RAGE and its other ligands S100P, S100A4, and HMGB-1 in human PDAC [84]. However, interaction of AGE with RAGE and their role in pancreatic cancer has not been explored in much detail. Prolactin is a peptide hormone and is secreted by the anterior pituitary gland. The closeness of this hormone with growth hormone and its functioning through tumour promoting Jak-STAT pathway strengthens the claim that prolactin has tumorigenic properties. The role of prolactin and prolactin receptor has been most well studied in breast and prostate cancer. Moreover, in hepatocellular carcinoma, colorectal cancer, ovarian cancer and endometrial cancer prolactin signaling has also been implicated [85]. Interestingly, prolactin signaling has also been shown to promote metastasis by inducing cell motility and also confers resistance to cancer cells to chemotherapeutic agents [86–89]. Furthermore, prolactin signaling has been found to facilitate pancreatic beta cell development and acinar cell growth [90]. However, its role in pancreatic cancer has not been discussed before and we report the involvement of prolactin signaling in pancreatic ductal adenocarcinoma, for the first time. Similarly, another interesting signaling pathway got selected was insulin resistance signaling pathway. Insulin resistance and Type II diabetes mellitus have been associated with different types of malignancy like hepatic, colorectal, breast, endometrial and also pancreatic [91]. Interestingly, the same IR and T2DM play a protective role in prostate cancer [92]. For quite some time there was a hypothesis linking IR to pancreatic cancer. Not much work has been done in that area until a recent study using a cohort of approximately 29,000 patients identified IR to be a risk factor for PDAC [93]. Hyperinsulinemia associated with IR and known mitogenic activity of insulin of insulin could be responsible for the process. Our results support this hypothesis and present with molecular proof behind the claim that IR predispose a patient with possibilities of developing PDAC. Therefore, our identification of conventional cancer related pathways as well as some interesting pathways not previously reported in PDAC, not only helps elaborating the cellular mechanism of action but also opens up avenues to interrogate and target key molecules in key altered pathways for better management of the disease in future.

Furthermore, as transcription factors are key factors bringing change in expression to a series of genes, TFs as miRNA targets are important for their ability to regulate expression of many genes simultaneously. We wanted to find out how many of our deregulated genes were transcription factors and careful investigation resulted in identification of 46 genes. As mentioned in their selection criteria, these genes have already been shown to be expressed in PDAC, evidenced mainly by high-throughput studies. Furthermore, we explored them in details for their functional significance in PDAC or in other cancers, if reported. HNF1B and MYBL2 were also reported to be down regulated in renal cell carcinoma, ovarian cancer and myeloid malignancies [94–96]. Unfolded protein response (UPR) is an important component of the endoplasmic reticulum (ER) stress and the most important aspect is to maintain the balance between cell death as a consequence and recovery from the stress [97]. ATF6 and XBP1 are two most important factors mediating the UPR and their downregulation could induce tumour cell death in aggressive cancers like PDAC, thereby acting as a regulatory mechanism in the overall process of tumourigenesis. Similar incident happened in case of HOXC11 too, where downregulation of this gene is known to suppress tumour formation and hence the phenomenon could be considered as body’s balancing act to check the tumour growth [98, 99]. Furthermore, there are no reports on the role of ESRRG, HES6 and NFIC transcription factors in PDAC. However, results from The Human Protein Atlas (https://www.proteinatlas.org/) shows poor survival outcome indicative of the disease aggressiveness associated with reduced expression of the gene in case of all of them [100]. Similarly, investigations on upregulated transcription factors identified members of Wnt, TGF-B, NF-kB signaling pathway which are known promoters of pancreatic carcinogenesis. Interestingly, we have identified several transcription factors like TRPS1, NFAT5, FoxF2, ELF4, RLF etc. which haven’t previously been reported to be involved in PDAC but shown to induce EMT, angiogenesis or proliferation of tumours in other organs. Among them, ELF4 has been found to be expressed during pancreatic development [101] and RLF overexpression has been correlated to poor survival in pancreatic cancer patients [100] strengthening the possibility of their involvement in the pathogenesis of PDAC, which could be explored further.

Our study also suffers from some limitations. For the separate meta-analyses of miRNAs altered in serum and tumour tissue, we focused only on studies where miRNA expression was investigated using microarrays. We excluded the small RNA sequencing results, thereby losing some probable candidates. However, when we looked at the small RNA sequencing results, we observed a large proportion of identified miRNAs with no experimentally validated targets at all. As our objective was to focus only on experimentally validated targets, we might not have missed out much of them concentrating only on microarray results. Furthermore, we couldn’t have combined both the analysis and small RNA sequencing datasets would have required separate meta-analysis altogether adding further complexity to the study. Another limitation, we feel, is lack of experiments to validate our finding in relevant cell lines or patient samples. The meta-analysis as well as the subsequent analysis has been performed with reasonable stringency and already got cross-platform validation results from miRNet and from PED. Above all, functional validation for most of the miRNAs belonging to the 21-miRNA meta-signature has already been done in pancreatic cancer, as evidenced by our extensive literature search. Hence, we excluded the experimental validation from this study and plan to perform them in the next one in much detail.

## Conclusion

Here, at first, we identify a 21 miRNA meta-signature of PDAC altered in tumour tissue and also secreted in serum. We further demonstrate that, apart from their possible role as biomarker, these miRNAs are also responsible for disease pathophysiology through deregulation of important pathways within the cell mediated by PDAC specific target genes.

## Supplementary information

**Additional file 1: Figure S1.** Network depicting miRNA-gene interactions encompassing the significantly enriched KEGG pathways; red circle denotes up-regulated genes, green circles denotes down-regulated genes and squares represents miRNA.

**Additional file 2: Table S1.** a: Information on miRNAs found to be upregulated in serum. This result is obtained after rank product calculation. b: Information on miRNAs found to be downregulated in serum. This result is obtained after rank product calculation.

**Additional file 3: Table S2.** a: Information on miRNAs found to be upregulated in pancreatic tumour tissue. This result is obtained after rank product calculation. b: Information on miRNAs found to be downregulated in pancreatic tumour tissue. This result is obtained after rank product calculation.

**Additional file 4: Table S3.** a: Information on experimentally validated gene targets of upregulated miRNAs in Pancreatic Cancer. This information is obtained using miRNet. b: Information on experimentally validated gene targets of downregulated miRNAs in Pancreatic Cancer. This information is obtained using miRNet.

**Additional file 5: Table S4.** List of all the miR-gene interaction pairs along with their fold change.

**Additional file 6: Table S5.** a: List of enriched GO terms in ‘GO Biological process’ as obtained by EnrichR using our genes of interest. b: List of enriched ‘KEGG Pathways’ as obtained by EnrichR using our genes of interest. c: List of enriched functions of genes as obtained by ‘GeneMANIA functions’ using our genes of interest.

**Additional file 7: Table S6.** List of transcription cofactors among our genes of interest.

## Data Availability

The data files generated during the current study have been submitted to GEO (Accession number: GSE140196). The relevant clinical information of the patients used in this study available upon reasonable request from the corresponding author.

## Abbreviations

*ADAM9*: ADAM Metallopeptidase Domain 9
BD: Becton Dickinson, USA
CLASH: Crosslinking, Ligation And sequencing of Hybrids
*CRK*: CRK Proto-Oncogene, Adaptor Protein
DEmiRs: Differentially expressed microRNAs
DETargets: Differentially Expressed Targets
*DUSP10*: Dual Specificity Phosphatase 10
EMT: Epithelial to Mesenchymal transition
FoxO: Forkhead Box O
GEO: Gene Expression Omnibus
GO: Gene ontology
HIF-1: Hypoxia Inducible Factor-1
IR: Insulin resistance
Jak-STAT: Janus Kinase- Signal Transducer And Activator Of Transcription
JNK: c-Jun N-terminal kinase
KEGG: Kyoto Encyclopedia of Genes and Genomes
*KRAS*: Kirsten Rat Sarcoma Viral Oncogene Homolog
LFC: Log fold change
miRNA: microRNA
mRNA: Messenger RNA
mTOR: Mechanistic Target Of Rapamycin Kinase
NF-kB: Nuclear Factor Kappa B
PAR-CLIP: Photoactivatable Ribonucleoside-enhanced Crosslinking and Immunoprecipitation
PI3K-Akt: Phosphatidylinositol-4,5-Bisphosphate 3-Kinase- AKT Serine/Threonine Kinase
qPCR: Quantitative Polymerase Chain Reaction
T2DM: Type 2 Diabetes Mellitus
TGF-b: Transforming Growth Factor Beta
TP53: Tumour Protein 53
