# Supplementary figures and images for: Comparison of tumour and serum specific microRNA changes dissecting their role in pancreatic ductal adenocarcinoma: a meta-analysis
Source: BMC Cancer. 2019 Dec 3;19:1175. doi: 10.1186/s12885-019-6380-z (PMC6891989; doi:10.1186/s12885-019-6380-z)

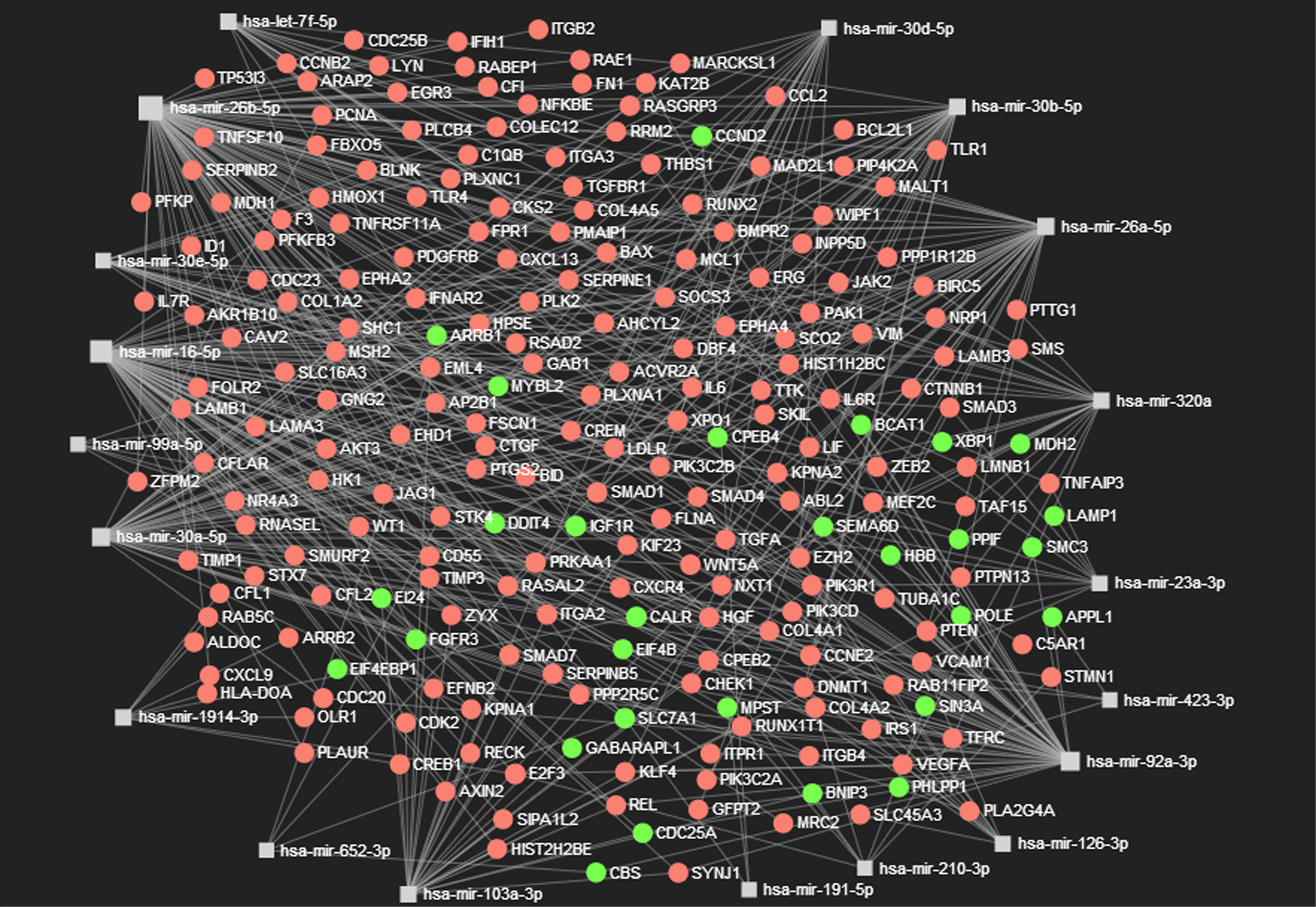

Supplement: Supplementary file 1 — Additional file 1: Figure S1. Network depicting miRNA-gene interactions encompassing the significantly enriched KEGG pathways; red circle denotes up-regulated genes, green circles denotes down-regulated genes and squares represents miRNA. [file 12885_2019_6380_MOESM1_ESM.tif]
